# Effect of oral fluconazole 1200 mg/day on QT interval in African adults with HIV-associated cryptococcal meningitis

**DOI:** 10.1097/QAD.0000000000001961

**Published:** 2018-09-19

**Authors:** Síle F. Molloy, John Bradley, Natasha Karunaharan, Muhammad Mputu, Neil Stone, Jacob Phulusa, Chimwemwe Chawinga, Kate Gaskell, Dalitso Segula, Damien Ming, Mary Peirse, Duncan Chanda, Shabir Lakhi, Angela Loyse, Cecilia Kanyama, Robert S. Heyderman, Thomas S. Harrison

**Affiliations:** a Centre for Global Health, Institute for Infection and Immunity, St George's University of London; b MRC Tropical Epidemiology Group, London School of Hygiene and Tropical Medicine, London, UK; c University Teaching Hospital, Lusaka, Zambia; d University of Zambia School of Medicine, Lusaka, Zambia; e University of North Carolina Project–Malawi, Kamuzu Central Hospital, Lilongwe; f Malawi–Liverpool–Wellcome Trust Clinical Research Programme, College of Medicine, University of Malawi, Malawi; g University College London, London, UK.; ∗ Joint first authors.

## Abstract

We assessed the effect of fluconazole 1200 mg/day on the QT interval in cryptococcal meningitis patients. Mean corrected QT (QTc) change from baseline to day 7 was 10.1 ms (IQR: −28 to 46 ms) in the fluconazole treatment group and −12.6 ms (IQR: −39 to 13.5 ms) in those not taking fluconazole (*P* = 0.04). No significant increase in QTc measurements over 500 ms was observed with fluconazole. Nevertheless, it remains important to correct any electrolyte imbalance and avoid concomitant drugs that may increase QTc.

Fluconazole remains in routine use as an induction regimen for the treatment of cryptococcal meningitis in many low and middle income countries, either alone, or in combination with amphotericin B. Guidelines recommend dosages of 800–1200 mg/day [1–3]. However, it has been previously suggested that fluconazole may prolong QT interval, either directly, or by inhibiting the hepatic metabolism of other QT-prolonging agents [4,5]. A lengthened heart rate-corrected QT (QTc) interval is a biomarker for ventricular tachyarrhythmias such as *torsades de pointes* and is a risk factor for sudden death, particularly in older patients [6]. In a prior study of cryptococcal meningitis, patients receiving amphotericin B with fluconazole (800 mg/day) had a slight increase in QTc compared with baseline (mean change from baseline: 6.6 ms (95% confidence interval −3.2 to 16.4 ms]) [4]. However, overall, day 7 QTc intervals for those treated with amphotericin B with fluconazole (800 mg/day) were similar compared with patients treated with amphotericin B in combination with fluconazole, at the lower dose of 400 mg/day, and amphotericin B alone; and there was no suggestion of an increase in the risk of clinically significant QTc prolongation (>500 ms) [4].

We assessed the effect of a fluconazole dose of 1200 mg/day, on QTc interval in a cohort of patients enrolled in the Advancing Cryptococcal meningitis Treatment for Africa (ACTA) trial. This was a phase III trial for the treatment of HIV-associated cryptococcal meningitis in Africa testing five different treatment regimens, three of which contained fluconazole at 1200 mg/day dosage [7]. ECGs to measure the QTc (using Bazett's formula) were performed for all participants at baseline and 1 week after enrolment, for the first 22 months of the study, until the Data Monitoring Committee recommended to discontinue routine ECG monitoring on the basis that the clinical risk did not warrant such monitoring in this context.

QTc results from patients randomized to regimens including fluconazole, 1200 mg/day (oral treatment of fluconazole with flucytosine for 2 weeks; amphotericin B with fluconazole for 1 week, and amphotericin B with fluconazole for 2 weeks, 2/3 of those enrolled in the trial) were compared with those randomized to non-fluconazole-containing regimens (amphotericin B with flucytosine for 1 week and amphotericin B with flucytosine for 2 weeks, 1/3 of those enrolled). Mean QTc length in each of the two groups at day 7 was compared by analysis of covariance (ANCOVA), adjusting for baseline QTc measurement. Mean change in QTc length from baseline to day 7 was analysed using a *t*-test and the proportion of patients with a long QTc (≥500 ms) in each group was compared at day 7 using Fisher's exact test. Analyses were performed using Stata, version 14.1 (StataCorp, College Station, Texas, USA).

A total of 150 patients had QTc results recorded at baseline with 104 (69.3%) randomized to a high-dose fluconazole treatment regimen. As expected, there was no significant difference in mean QTc length for patients randomized to fluconazole treatment compared with those randomized to no fluconazole at baseline (412.9 and 414.2 ms, respectively, *P* = 0.88). At baseline, 15 patients (10%) had Grade 1 (450–480 ms), six patients (4%) had Grade 2 (480–490 ms) and seven patients (4.67%) had Grade 3 QTc (>500 ms) at baseline. Of the patients with Grade 3 QTc at baseline, six resolved (<450 ms) by day 7 with electrolyte replacement and avoidance of known QT-prolonging concomitant drugs. Fluconazole was withheld temporarily for two patients. One patient with severe cryptococcal meningitis and sepsis died prior to a follow-up ECG.

Following commencement of antifungal treatment, QTc was recorded for 125 patients at day 7 with 84 (67.2%) randomized to high-dose fluconazole. Sixteen patients (12.8%) had Grade 1 (450–480 ms), eight patients (6.4%) had a Grade 2 (480–490 ms) and one patient (0.8%) had a Grade 3 QTc (>500 ms) at day 7. The patient with Grade 3 QTc had a normal QTc at baseline (437 ms) that increased at day 7 to 505 ms and this patient was randomized to a fluconazole-containing treatment regimen. The patient was well on discharge at day 14 of the study and completed 10-week follow-up without further adverse events. Overall mean QTc length at day 7 was 415.8 ms (IQR: 390–444). The mean change in QTc length from baseline was 2.9 ms (IQR: −30 to 37 ms): 10.1 ms (IQR: −28 to 46 ms) in the fluconazole containing treatment group compared with −12.6 ms (IQR: −39 to 13.5 ms) in those not taking fluconazole (*P* = 0.04; Table 1). There was evidence for a difference in mean QTc length for patients taking fluconazole treatment compared with those not taking fluconazole (422.5 and 402.1 ms, respectively, *P* = 0.01), adjusting for baseline QTc length. However, there was no evidence for a difference in the number of patients with long QTc (>500 ms) between the two groups at day 7 (one patient had a long QTc in the fluconazole group compared with none in the non-fluconazole group, *P* = 1.0; Table 1).

A small increase in mean QTc length for patients taking high-dose fluconazole (1200 mg/day) was observed in this study, as shown previously for doses of 800 mg/day [4]. As in the prior study, there was no evidence for an increase in the proportion of patients developing a clinically significant prolonged QTc interval. Since our study was conducted, preliminary results of a phase II trial using even higher fluconazole doses (1600 and 2000 mg/day) have been reported, without, as yet, details of QT interval effects but with no mention of serious concerns over QT interval prolongation [8].

Our study suggests that a fluconazole dose of 1200 mg/day does not lead to a clinically significant lengthening of the QTc interval and that ECG monitoring need not be mandatory in this population, treated at this dose for short periods. However, it is important to emphasize, as with all drugs with the potential to prolong QT interval, that it remains important to monitor and correct any electrolyte imbalance and to avoid, wherever possible, concomitant drugs that may also cause QTc prolongation.

## Acknowledgements

The main clinical trial was supported by grants from the Medical Research Council, United Kingdom (100504) and the French Agency for Research on AIDS and Viral Hepatitis (ANRS; ANRS12275). We would like to thank the patients involved in this study, as well as the staff at each site and the data and safety monitoring committee: Andrew Nunn, Halima Dawood, Andrew Kitua and William Powderly.

### Conflicts of interest

There are no conflicts of interest.

## Figures and Tables

**Table 1:** Corrected QT interval measurements at baseline and day 7 by treatment group.

	Day 7		Mean change from baseline to day 7 (*n* = 113)
	(*n* = 113)	(*n* = 125)	
Treatment group	Mean QTc, ms (IQR)	Long QTc, ms, *n* (%)	Mean change, ms (IQR)
Fluconazole	422.5 (401–448.5)	1 (1.0%)	10.1 (−28 to 46)
No fluconazole	402.1 (385–443)	0	−12.6 (−39 to 13.5)
*P* value	0.01^a^	1.0^b^	0.04^c^
Overall	415.8 (390–444)	1 (0.8%)	2.9 (−30 to 37)

IQR, interquartile range; QTc, corrected QT.

^a^ANCOVA analysis adjusting for baseline QTc.

^b^Fisher's exact test.

^c^*t*-test.
